# Intramolecular H-Bond Dynamics of Catechol Investigated by THz High-Resolution Spectroscopy of Its Low-Frequency Modes

**DOI:** 10.3390/molecules26123645

**Published:** 2021-06-15

**Authors:** Jonas Bruckhuisen, Guillaume Dhont, Anthony Roucou, Atef Jabri, Hamdi Bayoudh, Thi Thanh Tran, Manuel Goubet, Marie-Aline Martin-Drumel, Arnaud Cuisset

**Affiliations:** 1 UR4493, LPCA, Laboratoire de Physico-Chimie de l’Atmosphère, Université du Littoral Côte d’Opale, F-59140 Dunkerque, France; jonas.bruckhuisen@univ-littoral.fr (J.B.); guillaume.dhont@univ-littoral.fr (G.D.); anthony.roucou@uclouvain.be (A.R.); jabri_atef@yahoo.fr (A.J.); bayoudhhamdi@yahoo.fr (H.B.); thanhtay602@gmail.com (T.T.T.); 2 UMR8523—PhLAM—Physique des Lasers Atomes et Molécules, Université de Lille, CNRS, F-59000 Lille, France; manuel.goubet@univ-lille.fr; 3 Institut des Sciences Moléculaires d’Orsay, Université Paris-Saclay, CNRS, F-91405 Orsay, France; marie-aline.martin@universite-paris-saclay.fr

**Keywords:** catechol, intramolecular hydrogen bond, rovibrational spectroscopy, terahertz, low-frequency modes

## Abstract

Catechol is an oxygenated aromatic volatile organic compound and a biogenic precursor of secondary organic aerosols. Monitoring this compound in the gas phase is desirable due to its appreciable reactivity with tropospheric ozone. From a molecular point of view, this molecule is attractive since the two adjacent hydroxy groups can interchangeably act as donor and acceptor in an intramolecular hydrogen bonding due to the tunnelling between two symmetrically equivalent structures. Using synchrotron radiation, we recorded a rotationally-resolved Fourier Transform far-infrared (IR) spectrum of the torsional modes of the free and bonded -OH groups forming the intramolecular hydrogen bond. Additionally, the room temperature, pure rotational spectrum was measured in the 70–220 GHz frequency range using a millimeter-wave spectrometer. The assignment of these molecular transitions was assisted by anharmonic high-level quantum-chemical calculations. In particular, pure rotational lines belonging to the ground and the four lowest energy, vibrationally excited states were assigned. Splitting due to the tunnelling was resolved for the free -OH torsional state. A global fit combining the far-IR and millimeter-wave data provided the spectroscopic parameters of the low-energy far-IR modes, in particular those characterizing the intramolecular hydrogen bond dynamics.

## 1. Introduction

To a large extent, complex molecules in living matter have structures and properties that are determined by intramolecular hydrogen bonds (HBs). For understanding biological processes, the dynamics of HB formation and breaking is important [1]. For example, proteins are an important class of biomolecules where intramolecular HBs play a fundamental role in stability [2]. The rovibrational signatures of intramolecular HBs have mainly been studied in the mid-IR by probing the local stretching of the R-H donor and acceptor functional groups [3]. Yet, the vibrational modes directly involved in the dynamics of the intramolecular HB lie at lower frequencies in the far-IR region [4].

Compared to the mid-IR, the far-IR spectroscopy allows exploration of the dynamics of low-energy vibrations involving the full molecular backbone. That explains why far-IR/THz high-resolution spectroscopy is so efficient in discriminating subtle structural differences in particular in aromatic compounds (stable conformers [5], isomers [6], etc.). The low-frequency HB dynamics have been studied directly in the far-IR/THz domain with low or middle resolution vibrational spectroscopy techniques [7,8,9] or indirectly, at high resolution, using electronic excitations [10]. The full potential of high-resolution far-IR/THz gas-phase spectroscopy of isolated (bio)molecules with intramolecular HB remains unexplored today.

The main explanation for this statement is likely the difficulty to find a good molecular candidate and the adapted far-IR/THz spectroscopic technique allowing the detection and resolution of the rovibrational transitions involved in the intramolecular HB dynamics. Ortho-substituted aromatic compounds are probably the most simple and the most commonly cited examples of intramolecular HB. Among them, catechol (1,2-dihydroxybenzene) is an interesting molecule because its two vicinal hydroxy groups can act interchangeably as both hydrogen donors and acceptors in internal HB [11].

Moreover, the gas phase spectroscopic study of catechol is particularly interesting for at least two reasons: (i) catechol is produced in the atmosphere with high yields, around 80%, by the -OH oxidation of the major atmospheric aromatic compounds (benzene, phenol, cresol...) and its ozonolysis contributes significantly to the aerosol loading of the lower troposphere in polluted areas, where ozone concentrations could be high [12]; (ii) catechol moieties are also found widely within the natural world: drugs, hormones/neurotransmitters, poisons found in plants,... Many catechol derivatives have been suggested for therapeutic applications [13].

The microwave spectrum of catechol was studied at the end of the 1980s [11,14], and its assignment revealed a single conformation with a planar structure involving an intramolecular HB. The strength of this HB was discussed from the negative inertia defect, which arises mainly from out-of-plane vibrations of the molecule [15]. Only the ground state (GS) rotational transitions were assigned, and no spectroscopic features connected with the internal -OH rotations were observed.

The vibrations of catechol were also the subject of numerous IR and Raman studies in the gas phase and in the condensed phase. Gas phase investigations are limited to the spectral range above 740 cm^−1^, below this, only solid phase studies have been reported so far. The experimental frequencies were compared to the vibrational frequencies obtained from *ab initio* normal mode calculations at the HF/6-31G(d,p) level of theory [16].

The gas phase far-IR/THz spectra were measured recently by Bakker et al. using the free electron laser FELIX in a more general study of the low-frequency vibrational signatures of intramolecular HB in phenol derivatives [4,7]. The B3LYP–D3 functional provided the best results for the large amplitude anharmonic modes, such as the -OH torsional modes. Three vibrational modes have been identified, which are expected to be diagnostic for the HB strength: HB stretching and HB donating and accepting -OH torsion vibrations. For these measurements, the catechol molecules were expanded in a supersonic jet and probed via low-resolution conformer selective IR-UV ion dip spectroscopy (the REMPI technique).

In the present study, B3LYP and MP2 harmonic and anharmonic calculations using a large basis set were used to predict and interpret the high-resolution rotational and rovibrational spectra of catechol measured respectively by millimeter (mm)-wave and synchrotron-based Fourier-transform (FT)-far-IR spectroscopies. In addition to a fine description of the conformational landscape, a topological analysis of the intramolecular HB was performed.

To the best of our knowledge, the complete rovibrational analysis of the free and bonded torsional modes involved in the intramolecular HB presented in this study is unprecedented. Moreover, in addition to the refinement of GS parameters, the pure rotational transitions in the four lowest energy vibrationally excited modes were assigned in the mm-wave spectra. Finally, splittings due to tunnelling effects are resolved for the rotational transitions excited in the free -OH torsion state.

## 2. Results and Discussion

### 2.1. Quantum Chemistry Calculations

#### 2.1.1. Two-Dimensional Scan of -OH Torsional Modes

**Figure 1:**
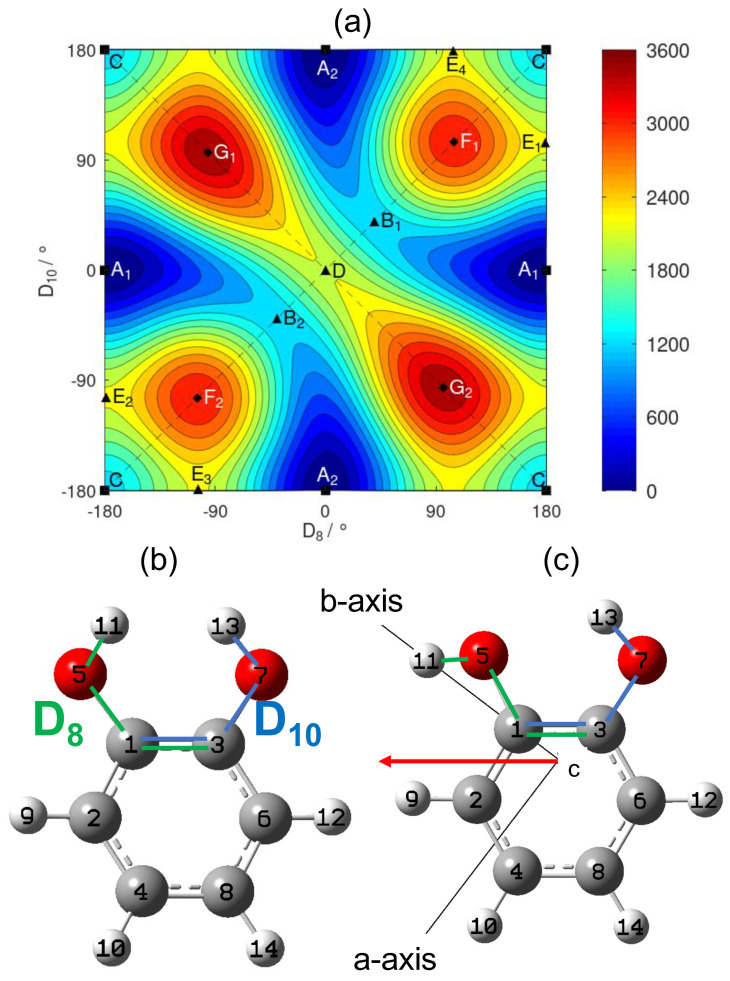
(**a**) 2D scan of the -OH torsional angles of catechol calculated with the B3LYP functional and the aug–cc–pVTZ basis set. The left and upper borders must be respectively identified with the right and lower borders. Contour curves are drawn every $200\;{\mathrm{cm}}^{-1}$. The points *A* to *G* correspond to stationary points of the two-dimensional surface, sorted by increasing energy, see Table 2. Symmetry equivalent points share the same letter and are distinguished by a subscript. Squares, triangles, and diamonds, respectively, indicate the minima, saddle points, and maxima. (**b**) Labelling of the nuclei of catechol and definition of the two torsional angles as the dihedral angles $D_{8}$ (green sequence of nuclei) and $D_{10}$ (blue sequence). This structure corresponds to the saddle point *D* (central point in the right figure) with $D_{8}=D\left(H(11)-O(5)-C(1)-C(3)\right)=0^{\circ }$ and $D_{10}=D\left(H(13)-O(7)-C(3)-C(1)\right)=0^{\circ }$. (**c**) Conformation $A_{1}$ corresponding to one of the two global minima. The permanent dipole moment in the (a,b) principal inertia plane is indicated by the red arrow.

In order to explore the conformational landscape of catechol, a 2D scan of the two -OH torsional modes was performed and is presented in Figure 1. The dihedral angles $D_{8}$ and $D_{10}$ used to describe the -OH torsional motions as defined in Figure 1 are different to those introduced in a previous 2D minimal energy torsional surface [17]. The authors in [18] highlighted the need to take care of the symmetry of the molecule in the definition of the angles involved in the description of the internal rotation.

The dihedral angles defined in Figure 1b allow a very convenient use of the symmetry of the catechol molecule, while the coordinates proposed by Bürgi et al. in [17] did not take advantage of it. The molecular symmetry group of catechol is $M_{S4}$, which was already used to interpret the structure of the energy levels in phenol [19]. In addition to the identity operation *E*, it contains the inversion operation $E^{*}$, the permutation $\left(\alpha \beta \right)$ which exchanges the nuclei with respect to the dashed vertical plane on Figure 1 and the permutation–inversion ${\left(\alpha \beta \right)}^{*}$. The action of the $M_{S4}$ group on the two torsional angles is given in Table 1.

The 2D scan in Figure 1 shows the electronic energy as a function of the two torsional angles. The surface reveals the existence of two equivalent global minima stabilized by an intramolecular HB. For minimum $A_{1}$$\left(\pm 180^{\circ },0^{\circ }\right)$, the O(5)-H(11) is the proton acceptor group and the O(7)-H(13) is the proton donor group, while the roles are reversed for the $A_{2}$ minimum $\left(0^{\circ },\pm 180^{\circ }\right)$. The path that starts from the global minimum $A_{1}$ and ends at the equivalent global minimum $A_{2}$ goes through the saddle points $B_{1}$ or $B_{2}$ whose energy is approximately $1200\;{\mathrm{cm}}^{-1}$ above the energy of the global minima. Point *C* corresponds to a secondary minimum that is significantly higher in energy (around $1300\;{\mathrm{cm}}^{-1}$) compared to minima $A_{1}$ and $A_{2}$.

The details of all the identified stationary points are presented in Table 2. Our energy surface depends on two dihedral angles $\left(D_{8},D_{10}\right)\in {[-180^{\circ },180^{\circ }[}^{2}$ and is, thus, defined on a two-dimensional torus. Therefore, we can check that the number of each type of stationary point is consistent with the topology of a torus. Using [20] and introducing the numbers $c_{0}=3$ of the minima ($A_{1}$, $A_{2}$, and *C*), $c_{1}=7$ of saddle points ($B_{1}$, $B_{2}$, *D*, $E_{1}$, $E_{2}$, $E_{3}$, and $E_{4}$) and $c_{2}=4$ of the maxima ($F_{1}$, $F_{2}$, $G_{1}$, and $G_{2}$), the equality $c_{0}-c_{1}+c_{2}=0$ indicates that we did not miss any stationary point.

#### 2.1.2. Topological Analysis of the Intramolecular H-Bond

**Figure 2:**
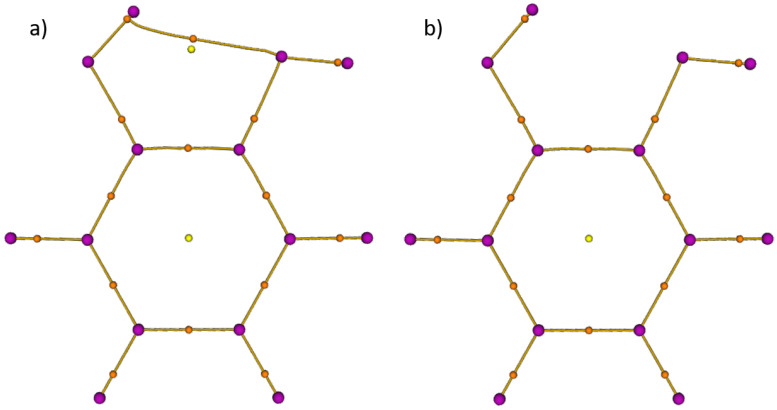
Topological analysis of the electron density of the global minimum *A* of catechol analysed by the Multifunctional Wavefunction Analyzer (Multiwfn) [22]. Purple, orange, and yellow dots are, respectively, the local maxima localized on the atoms, bond critical points, and ring critical points of the electron density. The B3LYP functional and different basis sets were used: (**a**) the intramolecular HB is visible with a cc–pVDZ basis set; (**b**) with diffuse functions and/or a larger basis set (aug–cc–pVDZ/cc–pVTZ/aug–cc–pVTZ), no intramolecular HB is visible.

The fingerprint of an intramolecular HB was searched in the electron density of the lowest energy conformer *A* (see Figure 1) of catechol calculated with the B3LYP functional. The topological analysis distinguishes the critical points of the electron density according to their signature. In particular, the nuclei are typically located at the maxima, while the bond critical points suggest the existence of either a covalent bond or a HB. Figure 2a presents the critical points determined with the cc–pVDZ basis set.

This shows the signature of an intramolecular HB. However, this one disappears in Figure 2b when a larger basis set, such as cc–pVTZ is used or when diffuse functions (aug–cc–pV*n*Z) are introduced. Figure 2a highlights that the cc–pVDZ overestimates the difference in the electron density between the HB and ring critical points. In [21], Mandado et al. noticed the same behaviour concerning the dependence on the size of the basis set. This dependence is due to the weak intramolecular HB of catechol compared to other phenol derivatives as was shown in [4].

### 2.2. Synchrotron-Based FT-Far-IR Spectroscopy

**Figure 3:**
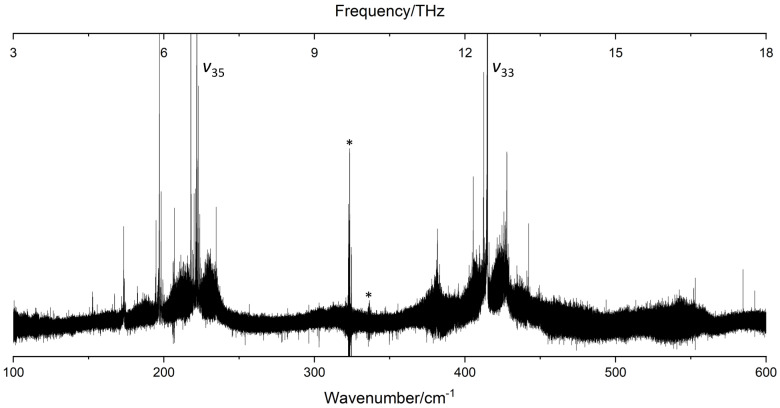
The FT-far-IR spectrum of catechol in the 100–600 ${\mathrm{cm}}^{-1}$ range measured at high-resolution ($10^{-3}\;{\mathrm{cm}}^{-1}$) at the AILES beamline of the SOLEIL synchrotron (630 scans co-added, P≃10μbar and T=300K). $\nu_{35}$ and $\nu_{33}$ label the bands associated with the free and bonded -OH torsional modes involved in the intramolecular HB. Artefacts due to bad compensations with the background spectrum are marked with an asterisk.

The synchrotron-based FT-far-IR spectrum of catechol, shown in Figure 3, was measured at high resolution (≃0.001 cm^−1^). In this section, we present, first, a vibrational analysis based on the Q-branches observed at medium and high resolutions. Then, we will describe the rovibrational fit of the two intense torsional -OH bands. To the best of our knowledge, it is the first time that a rovibrational assignment was performed on both free and bonded -OH torsional modes involved in the dynamics of the intramolecular HB.

#### 2.2.1. Vibrational Analysis

The catechol molecule belongs to the $C_{s}$ symmetry group with 36 normal modes: 25 A’ and 11 A” modes. In this study, we chose to keep the vibrational labelling of Gaussian output files with a classification based on the $C_{s}$ symmetry instead of the labelling based on the $C_{2v}$ symmetry of monosubstituted benzenes [23] used in previous catechol studies [16,17]. Table 3 presents the low-frequency vibrational modes lying in the far-IR spectral range shown in Figure 3. Ten fundamental vibrations are located below $600\;{\mathrm{cm}}^{-1}$: the antisymmetric $\nu_{36}$ and symmetric $\nu_{34}$ out-of-plane O-C-C-O twisting and wagging modes; the antisymmetric $\nu_{24}$ and symmetric $\nu_{25}$ in-plane O-C-C-O rocking and scissoring modes; the free $\nu_{35}$ and the bonded $\nu_{33}$-OH torsion; and four low-frequency ring deformations, $\nu_{32}$, $\nu_{31}$, $\nu_{23}$, and $\nu_{22}$.

Considering the theoretical calculations, the two modes associated to the free and bonded -OH torsion have strong intensities ($I_{\mathrm{harm}}>70\;{\text{km.mol}}^{-1}$) compared to the other low-frequency modes ($I_{\mathrm{harm}}<8\;{\text{km.mol}}^{-1}$). Indeed the FT-far-IR spectrum exhibits two intense rovibrational bands around $222\;{\mathrm{cm}}^{-1}$ and $415\;{\mathrm{cm}}^{-1}$, respectively, for the $\nu_{35}$ free and the $\nu_{33}$ bonded -OH torsion. These two bands are pure *c*-type, and their rotational structures are resolved in the P- and R-branches allowing the rovibrational analysis of catechol (see Section 2.2).

The measured vibrational band centres agree with the REMPI gas phase measurements of [4]. If we consider now the calculated harmonic and anharmonic IR frequencies and intensities at the B3LYP–D3/aug–cc–pVTZ level of theory, which provides us the best global agreement with the experimental ones, we note that the computational method fails to determine the anharmonic contribution of the -OH torsional modes. This could be explained by the large amplitude motions in these out-of-plane (oop) modes involved in the intramolecular HB, which cannot be treated with perturbation methods. These two torsional modes may be better described in a variational calculation with a Hamiltonian model of reduced dimensionality using our 2D PES [24].

Except for the two -OH torsional modes, all low-frequency fundamental vibrations of catechol exhibited poor IR activities with calculated intensities below $8\;{\text{km.mol}}^{-1}$. Yet, numerous other intense Q-branches were observed on both sides of the $\nu_{35}$ and $\nu_{33}$ band centres (see Figure 3). Considering the calculated frequencies and intensities, these Q-branches were not assigned to fundamental bands but to hot bands of the -OH torsional modes. None of the other modes have been measured yet in a gas phase far-IR spectrum except the $\nu_{25}$ mode, involving the intramolecular HB stretching, observed in the REMPI experiment of Bakker et al. [4] at $309\;{\mathrm{cm}}^{-1}$ in good agreement with our calculated values.

In our FT-far-IR spectra, no vibrational signature was visible in this region (see Figure 3). Compared to the *c*-type bands of the torsional modes, this A’ symmetry mode does not induce an out-of-plane variation of the dipole moment leading to a sharp Q-branch, which is easy to identify in the spectrum. The other O-C-C-O bending modes were observed only by solid state Raman measurements [16], and the measured frequencies are in the range defined by our B3LYP–D3 harmonic and anharmonic calculations. Finally, the overtones $2\nu_{36}$ and $2\nu_{35}$ and the combination band $\nu_{36}+\nu_{35}$ were predicted in the $400\;{\mathrm{cm}}^{-1}$ region by the anharmonic B3LYP–D3/aug–cc–pVTZ calculation. These bands are too weak to be observed and may be masked by the intense $\nu_{33}$ bonded -OH torsional band.

As mentioned previously, the numerous Q-branches, observed in the room temperature FT-far-IR spectra revealed complex vibrational patterns of hot bands on both sides of the $\nu_{35}$ and $\nu_{33}$ Q-branches. Their strong intensities reflect the strong *c*-type transition dipole between the GS and the two -OH torsional oop states. Some of these hot bands are localised far from the $\nu_{35}$ and $\nu_{33}$ fundamental vibrational band centres, especially in the blue side of $\nu_{35}$. This experimental observation assesses the strong anharmonicity of some modes confirmed by the anharmonic DFT calculations.

**Figure 4:**
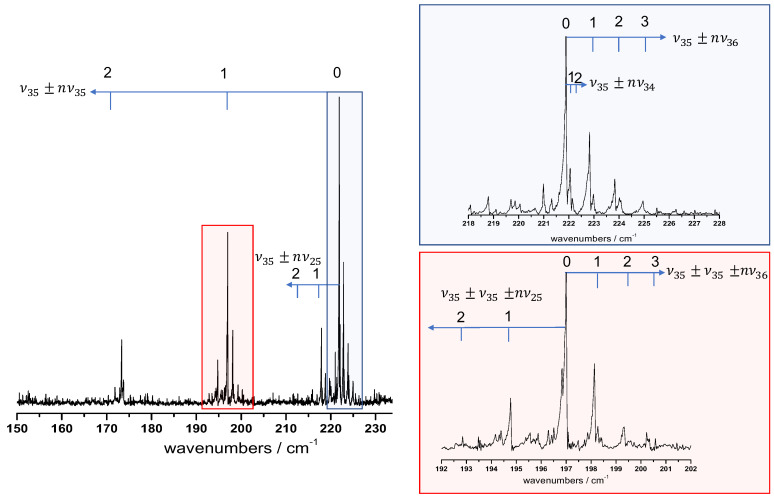
Hot band assignment proposition around the $\nu_{35}$ free -OH torsional band at middle resolution ($10^{-2}\;{\mathrm{cm}}^{-1}$). The assigned hot band progressions $\nu_{i}\pm n\nu_{j}$ and $\nu_{i}\pm \nu_{i}\pm n\nu_{j}$ are marked with blue arrows. Two spectral zones are zoomed: the blue panel shows the hot band sequence red shifted from the $\nu_{35}$ fundamental vibrational centre; the red panel highlights the hot band substructure around the $|2v_{35}\rangle \leftarrow |v_{35}\rangle$ vibrational transition.

In Figure 4, we propose a tentative assignment of the hot bands around the $\nu_{35}$ free -OH torsional band taking into account the four lowest energy vibrational states $\nu_{36}$, $\nu_{35}$, $\nu_{34}$, and $\nu_{25}$ that we know to be thermally excited due to the mm-wave analysis described in Section 2.3. First, we attempted to identify the four hot band sequences $|v_{35}+nv_{i}\rangle \leftarrow |nv_{i}\rangle$ (with i=36,35,34,25), labelled $\nu_{35}\pm n\nu_{i}$ in Figure 3, starting from the $\nu_{35}$ vibrational centre at $221.9\;{\mathrm{cm}}^{-1}$.

The assignment of the *i* mode to the $\nu_{35}\pm n\nu_{i}$ sequence was performed in two steps: (i) according to the Boltzmann factor, the fit of the exponential decay of integrated intensities between successive components of the hot band sequence provides an estimation of the energy difference between consecutive levels; (ii) anharmonic coefficients were deduced from the previous step and are compared with those obtained directly with the frequency progression of the hot band sequence.

Our hot band assignment suggested a large negative diagonal $x_{35,35}$ constant of about −12 cm^−1^, which constitutes an additional signature of the strong anharmonicity of the free -OH torsion. The off-diagonal anharmonic constants deduced from the three other $\nu_{35}\pm n\nu_{i}$ hot band sequences were smaller in absolute value with $x_{35,36}$, $x_{34,35}$ and $x_{33,35}$ estimated, respectively, about $+1\;{\mathrm{cm}}^{-1}$, $+0.2\;{\mathrm{cm}}^{-1}$, and $-4\;{\mathrm{cm}}^{-1}$. As was underlined previously, the anharmonic DFT calculation failed to evaluate the anharmonicity of the -OH torsions, which prevents a reliable comparison between the experimental and computed $x_{i,35}$ constants.

Afterwards, we noticed that the pattern of these hot band sequences observed around the $\nu_{35}$ band centre was repeating in each component of the strongly blue-shifted $\nu_{35}\pm n\nu_{35}$ sequence. Furthermore, we observed hot band sequences starting from combination levels. An example is given in the red panel of Figure 4 with two sequences, one blue-shifted ($\nu_{35}\pm \nu_{35}\pm n\nu_{25}$) and one red-shifted ($\nu_{35}\pm \nu_{35}\pm n\nu_{36}$) from the $\nu_{35}\pm \nu_{35}$ origin.

Finally, for a complete hot band assignment, including $\nu_{33}$, a specific study should be performed in a future article in order to confirm and finalize our present work. As was done for thermally excited molecules, such as benzene [25] or naphthalene [26], a suitable treatment based on an anharmonic force field would have to be carried out for the hot bands rovibrational assignment. In the case of catechol with a high number of low-energy states, the possibility of resonances between close-lying states should be considered, and, in addition to the anharmonic force field calculation, the multi-state interaction problem should be solved by considering numerous Coriolis/Fermi/Darling–Dennison couplings to have a better prediction of the hot band sequences.

#### 2.2.2. Rovibrational Analysis

**Figure 5:**
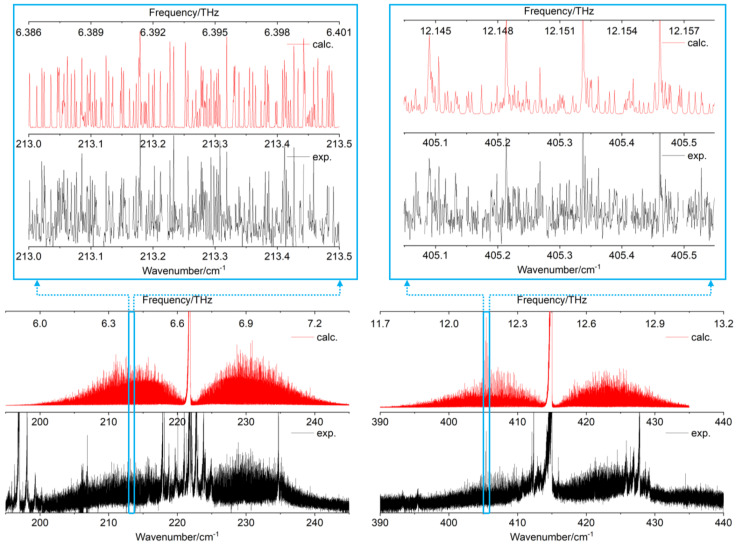
**Left**: rovibrational band of the $\nu_{35}$ free -OH torsional mode. **Right**: rovibrational band of the $\nu_{33}$ bonded -OH torsional mode. Experimental synchrotron-based FT-far-IR spectra are shown in black. Simulated spectra with the fitted constants of Table 4 are presented in red. Two zoomed parts highlight the quality of the rovibrational fit in the P-branches of the two fundamental torsions.

In our rovibrational analysis, we treated the $|v_{35}=1\rangle$ and $|v_{33}=1\rangle$ as isolated vibrational states, i.e., we considered no explicit Coriolis interactions and no vibrational resonances with other close lying states. This turned out to be sufficient considering the accuracy of our data and the range of the observed *J* values. The fitted parameters in the far-IR analysis of the $\nu_{35}$ and $\nu_{33}$ bands are presented in Table 4, and the simulated far-IR spectra with these parameters are compared to the experimental ones in Figure 5.

The two experimental rovibrational patterns shown on a spectral range of 50 cm^−1^ (Figure 5, black curves) exhibit several hot bands, which overlap the P, Q, and R branches of the fundamentals. In this work, the rovibrational lines belonging to hot bands were not included in the fit (see Figure 5, red curves) and numerous rovibrational lines remain unassigned (see Figure 5, zoomed parts). Nevertheless, more than 11,000 rovibrational *c*-type transitions were assigned in the FT-far-IR spectrum (6937 for $\nu_{35}$ and 4131 for $\nu_{33}$) highlighting the highly congested far-IR spectrum of catechol.

Numerous rovibrational lines are blended, especially between quasi-degenerate transitions with $K_{a}^{\prime }=K_{a}^{\prime \prime }\in \left\{0,1\right\}$ or $K_{c}^{\prime }=K_{c}^{\prime \prime }\in \left\{0,1\right\}$. Therefore, the number of fitted transitions is larger than the number of fitted frequencies. A development of the centrifugal distortion up to quartic terms in the effective Hamiltonian was sufficient to reproduce the experimental transition frequencies of the two bands $\nu_{35}$ and $\nu_{33}$ at the experimental accuracy with an unitless RMS close to 1.1.

All the molecular parameters for the two torsional bands were determined with a high degree of accuracy. In particular, the vibrational band centres of $\nu_{35}$ and $\nu_{33}$ were fitted with an accuracy better than 10^−4^ cm^−1^, four orders of magnitude better than those obtained with the REMPI technique in [4,7]. The complete set of rotational and centrifugal distortion constants were perfectly determined, and the detailed fit is given in the Supplementary Materials.

From the results of Table 4, we determined the differences between the GS rotational constants and those in the $|v_{35}=1\rangle$ and $|v_{33}=1\rangle$ torsionally excited states (ES) according to the relation $\alpha_{i}^{A}=A_{GS}-A_{i}$ with i=32,35 and similarly for the other rotational constants *B* and *C*. The vibration-rotation constants $\alpha_{i}$ allow for estimation of the influence of the two torsional modes on the overall rotation of the catechol molecule.

From the fitted values of Table 4 for the free -OH torsional mode, we obtained $\alpha_{35}^{A}=2.2146\;\mathrm{MHz}$, $\alpha_{35}^{B}=2.4306\;\mathrm{MHz}$, and $\alpha_{35}^{C}=0.0063\;\mathrm{MHz}$. Similarly, for the bonded -OH torsional modes, the values were $\alpha_{33}^{A}=8.6671\;\mathrm{MHz},\;\alpha_{33}^{B}=0.0883\;\mathrm{MHz}$, and $\alpha_{33}^{C}=0.56801\;\mathrm{MHz}$. While the rotation of catechol in the ring plane around the c-axis is almost not affected by the -OH torsional modes, we observed significant decreasing of the $A_{35}$, $B_{35}$, and $A_{33}$ rotational constants.

With a bonded -OH almost parallel to the b-axis (see Figure 1, right part), the largest vibration-rotation constant $\alpha_{i}$ was obtained for a rotation around the a-axis ($\alpha_{33}^{A}≃8.7\;\mathrm{MHz}$). The free -OH torsion $\nu_{35}$ also perturbed the overall rotation around the a- and b-axes but with a lesser influence compared to $\nu_{33}$ ($\alpha_{35}^{A,B}≃2.3\;\mathrm{MHz}$). Here, we observe that the free and the bonded -OH torsions differently affected the overall rotation of the catechol molecule.

### 2.3. Millimeter-Wave Rotational Spectroscopy

**Figure 6:**
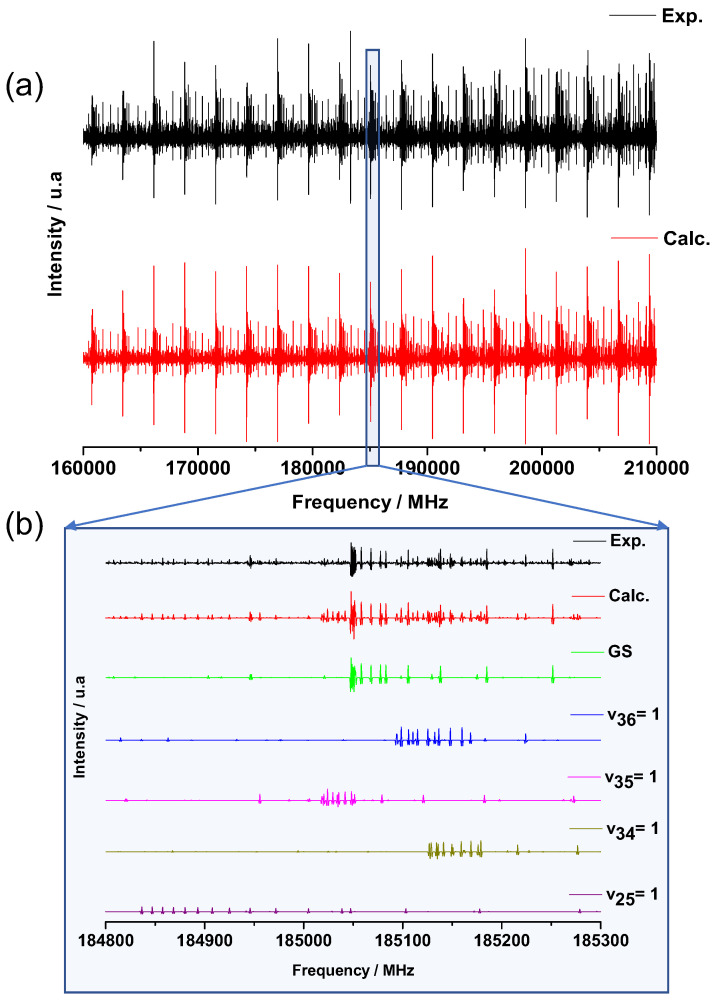
(**a**) Experimental (black) and calculated (red) rotational spectrum of catechol in the 160–210 GHz frequency range. (**b**) Zoom on a 500 MHz range highlighting the GS and the different low-frequency vibrationally ES contributions to the calculated spectrum.

Using the subTHz spectrometer (see Section 3.2.2), the room temperature Doppler limited mm-wave pure rotational spectra of catechol were recorded in the 70–110 GHz and 140–220 GHz regions. The spectra are highly congested with most of the measured rotational lines blended. Up to eight transitions can be associated to one line. In addition to the GS, four low frequency vibrationally ES were populated sufficiently at room temperature leading to the dense spectrum of Figure 6a. The enlarged 500 MHz region of Figure 6b illustrates how the spectra are composed of these five vibrational states and can be reproduced by our simulation (red curve Figure 6) at the experimental accuracy. Only a negligible number of weak lines remains unassigned.

#### 2.3.1. Ground State Analysis

The analysis of the mm-wave spectra was started with a line-by-line assignment of the GS transitions (see Section 3.2.2). Based on the molecular constants of Caminati et al. [11] and including 171 cm-wave lines of their study, almost 10,000 new transitions were assigned in the present work for the GS, associated with more than 4600 mm-wave lines. Among the assigned transitions, 75.8% were of *b*-type and 24.2% were of *a*-type. According to the permanent dipole orientation in the (a,b) plane (see red arrow in Figure 1), the *b*-type lines were stronger than the *a*-type ones.

Our results indicate that the ratio $\mu_{b}/\mu_{a}$ is probably underestimated by the B3LYP–D3/aug–cc–pVTZ calculations $\mu_{b}/\mu_{a}≃1.4$ and overestimated by the approximation made with the sum of the dipole moments of phenol $\mu_{b}/\mu_{a}≃9.2$ as was done in [14]. All the determined parameters with their uncertainties are listed in Table 5.

The huge number of catechol GS rotational lines are reproduced at the experimental accuracy with a RMS of 55kHz and a unitless RMS of 1.17. The fitted spectroscopic parameters include three rotational and nine centrifugal distortion constants. Compared to the previous GS cm-wave analyses [11,14] including GS rotational transitions with $J_{\max}<50$, the fit of this mm-wave study was extended to $J_{\max}=124$ requiring to add four sextic centrifugal distortion terms in the asymmetric top effective Hamiltonian: $\Phi_{KJ}$, $\phi_{J}$, $\phi_{K}$, $\phi_{JK}$ determined for the first time.

The fitted GS rotational constants allow an accurate determination of the inertia defect ΔI, which is a measure of the non-planarity of the catechol molecule. Despite its equilibrium planar structure, the small negative value arises mainly from the quantum zero-point motion of the oop low-energy vibrations detailed in Table 3 [14,15].

#### 2.3.2. Vibrationally Excited State Analysis

A total of 64% of the assigned transitions in the global fit of the mm-wave catechol spectra belong to thermally excited vibrational states. Pure rotational transitions in four different vibrationally ES have been identified, and the Table 5 lists the four sets of fitted molecular parameters. For each of them, rotational and centrifugal distortion constants developed up to quartic terms were determined with a high degree of accuracy. Only a slight degradation of the RMS was observed for the fit of the $|v_{25}=1\rangle$ ES due to the weak intensity of its rotational lines (see Figure 6b). For higher energy far-IR vibrations, such as the bonded -OH torsion $\nu_{33}$, the pure rotational transitions were too weak to be observed in the room temperature mm-wave spectra.

The most intense mm-wave lines, which do not belong to the GS, were assigned to the lowest energy $|v_{36}=1\rangle$ ES associated to the O-C-C-O twisting not observed in the far-IR spectrum. As expected, we obtained the largest negative value for the inertia defect for the lowest energy oop vibration (see Table 5).

**Figure 7:**
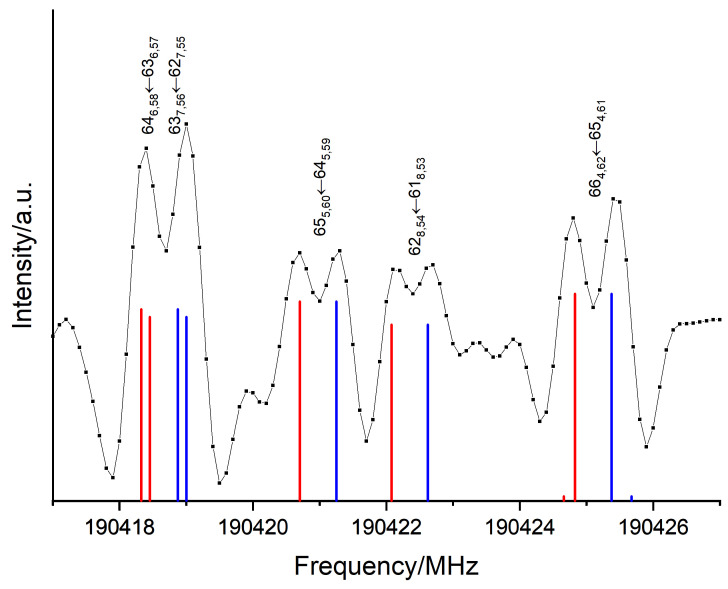
Tunnelling splittings observed in the pure rotational mm-wave spectrum within the $|v_{35}=1\rangle$ ES. Each doublet associated with $J_{K_{a}^{\prime },K_{c}^{\prime }}^{\prime }\leftarrow J_{K_{a}^{\prime \prime },K_{c}^{\prime \prime }}^{\prime \prime }$ rotational transitions represented by their blue and red components.

The $\nu_{35}$ parameters fitted from the FT-far-IR measurements in Table 4 were used as an initial set of parameters to start the assignment of the rotational transitions in the $|v_{35}=1\rangle$ ES associated with the free -OH torsion. We found doublets separated by around 600kHz around each predicted line, as depicted in Figure 7. Such doublets were already observed in phenol and associated to the torsion of its single -OH group yielding a double minimum potential. The $V_{2}$ tunnelling barrier separating the two symmetrically equivalent structures of phenol was about $1200\;{\mathrm{cm}}^{-1}$ [19]. In this case, splittings larger than 56MHz were observed by L. Kolesniková et al. in the phenol mm-wave rotational spectrum both within the GS and within the -OH torsion ES [27] and by S. Albert et al. in the phenol far-IR rovibrational spectrum of the torsional fundamental [19].

In Section 2.1, our results showed that the height of the saddle point *B* separating the two global minima $A_{1}$ and $A_{2}$ (see Figure 1a) was calculated to be about $1200\;{\mathrm{cm}}^{-1}$, as in phenol. This corresponds to the tunnelling barrier of the -OH torsions separating the two equivalent conformers. The splittings in the $|v_{35}=1\rangle$ torsional state of catechol are two orders of magnitude smaller than in the first excited torsional state of phenol. This can be explained by a higher -OH internal rotation barrier in catechol associated to a path from the minimum $A_{1}$ to $A_{2}$ requiring a concerted motion of the two hydroxy groups.

Due to the regular splitting, it was possible to analyse both tunnelling sublevels $|v_{35}=1^{+}\rangle$ and $|v_{35}=1^{-}\rangle$ simultaneously following $H_{\mathrm{eff}}=\Delta E+H_{\mathrm{rot}}\left(+\right)+H_{\mathrm{rot}}\left(-\right)$ without implementing any additional terms. Both stacks of rotational sublevels were simultaneously fitted. The resulting sets of parameters were almost identical with differences smaller than the uncertainties (indicated by the blue and red values of Table 5). The splittings of the energy levels created doublets of lines separated by 2ΔE. A total of 1651 *a*-type and 3309 *b*-type transitions up to $J_{\max}=99$ were assigned for both sublevels leading to a RMS of 99 kHz, a unitless RMS of 0.93 and ΔE= 312 kHz. This small splitting of about 600 kHz on average was not resolvable in the FT-far-IR of Section 2.2; hence, it was not observed.

Lying in the 300 cm^−1^ region (see Table 3), the $|v_{34}=1\rangle$ and $|v_{25}=1\rangle$ ES are sufficiently populated to be also observed in the mm-wave rotational spectra. Two independent sets of molecular constants were fitted and non-ambiguously assigned to the antisymmetric O-C-C-O wagging oop $\nu_{34}$ and symmetric scissoring in-plane (ip) $\nu_{25}$ vibrations due to the sign of their inertia defects. Indeed, compared to the other A” ES, the fitted rotational constants of the $|v_{25}=1\rangle$ ES yielded a positive inertia defect reflecting the A’ symmetry of the ip intramolecular HB stretching. A remarkable agreement between the calculated anharmonic inertia defects $\Delta I_{\mathrm{calc}}$ and the experimental ones ΔI is observed in Table 5 except for the $|v_{34}=1\rangle$ ES, where the anharmonic calculation underestimates the inertia defect involved by the O-C-C-O wagging.

Finally, Table 6 summarises the vibrational band centres ν and inertia defects ΔI of phenol and catechol provided respectively in [27] and in this study (see Table 5) for the lowest energy oop vibrations. In this table, we calculate a “GS corrected” inertia defect $\Delta I_{i}^{*}$ defined by the difference between the inertia defect $\Delta I_{i}$ in the vibrationally ES and the inertia defect $\Delta I_{GS}$ in the GS. In first approximation, $\Delta I_{i}^{*}$ is given by:

$$\Delta I_{i}^{*}=\Delta I_{i}-\Delta I_{GS}≃\frac{h}{8\pi^{2}}\left(\frac{\alpha_{i}^{C}}{C_{GS}^{2}}-\frac{\alpha_{i}^{B}}{B_{GS}^{2}}-\frac{\alpha_{i}^{A}}{A_{GS}^{2}}\right).$$

$\Delta I_{i}^{*}$ indicates the effective contribution of the low-frequency vibrational excitation on the inertia defect of the molecule. Table 6 compares the frequency and the inertia defects of the C-O and -OH oop bending modes in the case of a single hydroxy group and in the case of two hydroxy groups connected by a weak intramolecular HB.

From this comparison, three remarks may be made with respect to the HB dynamics:

(i) Instead of a single oop C-O bending in phenol, we observed, in catechol, one blue-shifted vibration (the O-C-C-O wagging) and one red-shifted vibration (the O-C-C-O twisting). This statement also applies to the -OH torsions with one blue-shifted frequency (the bonded -OH) and one red-shifted frequency (the free -OH) due to the local anisotropy visible on the 2D PES around $A_{1}$, see Figure 1. The -OH group, which acts as HB donor, leads to a blue-shift of the associated torsional mode frequency in the far-IR domain [7]. It constitutes one of the signatures of the intramolecular HB formation [2].
(ii) The intramolecular HB is preserved in the in-phase wagging of the two hydroxy groups, while it is perturbed in the out-of-phase twisting. This perturbation seems to increase the inertia defect $\Delta I_{36}$ of catechol compared to the ones of phenol and $\Delta I_{34}$ of catechol.
(iii) Compared with phenol, $\Delta I^{*}$ of catechol was found to be larger (in absolute value) for the free -OH and smaller for the bonded -OH. This is a contrary behaviour compared to the one observed in (i) with the vibrational frequencies. There is a specific effect of stabilization due to the intramolecular HB: the torsion of the -OH group, which acts as HB donor has a smaller contribution to the inertia defect than the torsion of -OH group, which acts as an HB acceptor.

## 3. Materials and Methods

### 3.1. Theoretical Methods

#### 3.1.1. Quantum Chemistry Methods

The quantum chemistry calculations were performed with the Gaussian 16 package [28]. The DFT calculations were performed with the B3LYP [29] and B3LYP–D3 [30] functionals with the default ultrafine grid and compared with results from MP2 theory in order to evaluate the efficiency of different functionals and methods for the prediction of the low frequency vibrations. The calculations were performed with the correlation consistent basis set aug–cc–pVTZ [31].

The triple zeta quality is known for its reliability with comparable molecules [15], and the diffuse functions help for the description of the intramolecular HB (see Section 2.1.2). The geometry of the catechol molecule has been represented with a *Z*-matrix that explicitly makes use of the torsional angle of the two -OH groups. The optimisation of the structures was performed with the tight criterion. The energy landscape of the two torsional modes of the catechol molecule in its electronic GS was determined by quantum chemistry calculations. A two-dimensional scan was obtained after a series of molecular geometry optimizations.

Each optimization released all the internal coordinates except for the two torsional angles *D*_8_ = *D*(H(11)-O(5)-C(1)-C(3)) and *D*_10_ = *D*(H(13)-O(7)-C(3)-C(1)) of Figure 1, which were kept frozen. The scan step is $5^{\circ }$ for both torsional angles. The topology of the electron density was analysed by the Multifunctional Wavefunction Analyzer (Multiwfn) [22]. The harmonic and anharmonic vibrational frequencies and intensities of the lowest energy conformer were computed with the B3LYP and B3LYP–D3 functionals.

We used the anharmonic calculations implemented in Gaussian 16 with the option freq=anharm, which numerically computes the third and fourth derivatives of the electronic energy at the global minimum. The anharmonic calculations provided us an estimation of the rotational constants in the vibrationally ES (see Table 5) and of the anharmonic coupling parameters useful for the hot band assignment (see Section 2.2.1).

#### 3.1.2. Effective Hamiltonians

For the assignment of the high resolution FT-far-IR rovibrational and mm-wave rotational spectra, Pickett’s programs SPFIT/SPCAT [32] were used. A Watson quasi-rigid rotor effective Hamiltonian in the *A* reduction [33] in $I^{r}$ representation was developed up to sextic centrifugal distortion constants for the vibrational GS and up to quartic for the ES.

The initial predictions of rovibrational frequencies were performed using the GS rotational parameters from the microwave study of [11] and the measured band centres of the strongest Q-branches observed in the 222 and $415\;{\mathrm{cm}}^{-1}$ regions (see Figure 5). The variation of the rotational constants between the GS and the ES $\nu_{35}$ and $\nu_{33}$ was evaluated by multiplying the anharmonic B3LYP/aug–cc–pVTZ rotational constants with a scaling factor corresponding to the ratio between the experimental ([11]) and calculated GS values. This method obtains an initial set of rotational constants with a relative accuracy better than 1% [34]. The rovibrational bands $\nu_{35}$ and $\nu_{33}$ of the FT-far-IR spectra were assigned using the Loomis–Wood assignment procedure for asymmetric top molecules with the LWWa software [35].

For the subsequent mm-wave analysis of Section 3.2.2, the fitted GS parameters of [11] were re-evaluated with the mm-wave measurements. The uncertainty of each line was adjusted between 10 and 300 kHz in the weighted fit performed with SPFIT using Equation (2) of [36] taking into account the estimated S/N ratio of our measured mm-wave lines. The SVIEW and ASCP software of the AABS package of Kisiel [37] were used to directly assign the measured lines to predicted rotational transitions. The fit procedure for the pure rotational transitions in the low frequency vibrational ES was performed using initial sets of constants determined with the same scaling approach used for the rovibrational analysis.

### 3.2. Experimental Methods

The FT-far-IR rovibrational and the mm-wave rotational spectra were measured, respectively, with the IFS125HR Bruker interferometer coupled to the AILES THz/far-IR beamline of the SOLEIL synchrotron [38] and with the solid state subTHz source of the LPCA in Dunkirk [39]. Solid catechol with a stated purity of 99% was purchased from Fisher Scientific. The room temperature equilibrium vapour pressure (P(300K)≃1Pa [40]) was directly injected in the absorption cells without further purification. The FT-far-IR and mm-wave measurements were performed, respectively, in static and controlled flow conditions.

#### 3.2.1. Synchrotron-Based FT-Far-IR Measurements

The synchrotron radiation was extracted and focused onto the entrance aperture of the Bruker IFS 125 FT interferometer equipped with a 6µm mylar–silicon composite beamsplitter suitable for the THz/far-IR spectral range. A total absorption path length of 150 m was reached by using a White–type multipass cell, which was isolated from the interferometer by 50µm thick polypropylene windows. The far-IR/THz signal was detected in the 10–700 ${\mathrm{cm}}^{-1}$ spectral range with a helium-cooled silicon bolometer equipped with an optical band-pass filter.

The rovibrational spectra shown in Section 2.2 were recorded at the maximum resolution of the instrument ($R=0.00102\;{\mathrm{cm}}^{-1}$), where the rovibrational linewidth is limited by the apparatus function (≃30 MHz). More than 600 scans were co-added allowing the observation of all the far-IR active fundamental vibrational bands (see Table 3).

The corresponding acquisition time was close to 50 h. We estimated that, using a classical source, such as a Hg lamp, 75 days of continuous acquisition would have been required to obtain an equivalent S/N ratio in the $\nu_{35}$ region (see Figure 5a) [38]. Each spectrum was calibrated using residual water absorption lines, whose wavenumbers were taken from [41], and the experimental error on the rovibrational line wavenumbers included in the fitting procedure was estimated at $0.0002\;{\mathrm{cm}}^{-1}$.

#### 3.2.2. Millimeter-Wave Measurements

The mm-wave absorption spectra in the 70–220 GHz shown in Section 2.3 were recorded at room temperature with the solid state subTHz spectrometer developed in the LPCA laboratory and described in detail in [39]. The measurements were performed in flux conditions at P=1.5Pa at the Doppler broadening limit of the rotational lines ($\Delta \nu_{\mathrm{Doppler}}∼$ 150 kHz at ν≃ 100 GHz and T≃300 K). The source is an amplified multiplication chain from Virginia Diodes Inc., which up-converts a synthesized microwave frequency.

The guided radiation was launched into free space using a horn antenna and propagated through a 125-cm long and 5.6-cm diameter stainless steel absorption cell closed by two Teflon windows. Using a polarization grid and a roof-top reflector, the interaction path-length was doubled [42]. A pair of off-axis parabolic mirrors were used to collimate the radiation into the cell and to refocus it subsequently onto a Zero Biased Detector, which was an unbiased Schottky diode mounted in a wave-guide operating in detection mode. The spectra have been recorded using 100 kHz frequency steps and a time constant of 100 ms.

The 2F modulation detection scheme was used with a frequency of 13 kHz and depths of 270 kHz and 540 kHz, respectively, in the 70–110 GHz and the 140–220 GHz ranges. A band-pass filter was applied to the spectra cutting off low frequencies and the noisy high frequency part. By this post treatment, the noise level was slightly decreased, and the low frequency baseline variations caused by stationary waves between the source, the polarization grid, the cell, and the detector were removed as was previously done in [6].

## 4. Conclusions

The dynamics of an intramolecular HB were investigated through the high-resolution THz rovibrational spectroscopy of the low-frequency modes of catechol in gas phase. In addition to its biological and environmental impacts, catechol is an excellent prototype to study the intramolecular HB dynamics since its two vicinal hydroxy groups can act interchangeably as both hydrogen donors and acceptors. The 2D PES of catechol built by scanning the two -OH torsions showed local anisotropy around the lowest energy equilibrium structures stabilized by a weak intramolecular HB.

We performed synchrotron-based FT-far-IR spectroscopy to explore the influence of the HB on the dynamics of the nuclei. The rovibrational spectrum revealed two far-IR active bands $\nu_{35}$ and $\nu_{33}$ associated, respectively, with the -OH free and bonded torsions. Since the spectrum was recorded at room temperature, numerous *c*-type hot bands were observed around these two fundamental bands, and a proposition of assignment was conducted around $\nu_{35}$. Hot bands involving the four lowest energy vibrational states were identified with signatures of strong anharmonicities, which cannot be reproduced by our quantum chemistry calculations based on perturbative methods.

A rovibrational assignment of the two torsional modes involving the -OH groups was performed for the first time. The comparison of the fitted molecular parameters of the $|\nu_{35}=1\rangle$ and $|\nu_{33}=1\rangle$ states highlights the different influences of the free and bonded -OH torsions on the overall rotation of catechol. Using a solid state subTHz spectrometer, the room temperature Doppler limited mm-wave pure rotational spectrum was also recorded in the 70–220 GHz region. The GS parameters were refined, and pure rotational transitions in the four lowest energy vibrationally ES were assigned. Very small tunnelling splittings were observed in the $|\nu_{35}=1\rangle$ ES.

Finally, the inertia defects were determined with a high degree of accuracy, confirming the vibrational assignment to the different ES of the mm-wave transitions. As an example, the experimental inertia defect of the intramolecular HB stretching $\nu_{25}$ was the only positive one, and this is consistent with the theoretical prediction. For the other ES, the influence of the low-frequency vibrational dynamics on the catechol planarity was discussed by comparing the negative inertia defects.

In particular, a specific effect of stabilization due to the intramolecular HB was observed with the free -OH torsion, which contributes more strongly to the inertia defect compared to the bonded -OH torsion. While the vibrational frequency measurements of the free and bonded -OH stretching and torsion were used in previous low-resolution studies to directly probe the intramolecular HB, high-resolution rovibrational THz spectroscopy provides a new perspective by investigating the influence of the low-frequency vibrational modes on the overall rotation of systems with intramolecular HB.

Some molecular parameters accessible by rotational spectroscopy, such as the inertia defect, may be determined with a high degree of accuracy and may be used as an alternative probe of the HB strength [14]. Therefore, we propose, as was done by Bakker et al. in [4,7] with REMPI far-IR low-frequency vibrational measurements and Born–Oppenheimer Molecular Dynamics calculations, a larger study on different phenol derivatives showing intramolecular HB with different HB strengths.

In this aim, a double challenge experimental and theoretical has to be overcome by resolving rotationally the low-frequency vibrational bands involving stronger intramolecular HB and by performing variational calculations that are able to provide better descriptions of the anharmonicities in the large amplitude motions and of the resonances between the different modes.

## Figures and Tables

**Table 1:** The transformation properties of the dihedral angles $D_{8}$ and $D_{10}$ under the elements of the $M_{S4}$ molecular symmetry group.

	*E*	$E^{*}$	$\left(\alpha \beta \right)$	${\left(\alpha \beta \right)}^{*}$
$D_{8}$	$D_{8}$	$-D_{8}$	$D_{10}$	$-D_{10}$
$D_{10}$	$D_{10}$	$-D_{10}$	$D_{8}$	$-D_{8}$

**Table 2:** Stationary points of the energy surface of Figure 1. The B3LYP and MP2 relative energy calculations were performed with the aug–cc–pVTZ basis set. The position of the other points labeled on the Figure 1a can be deduced from symmetry arguments and Table 1.

Point	$D_{8}{/}^{\circ }$	$D_{10}{/}^{\circ }$	Stationary Point	$\Delta E_{\mathrm{B3LYP}}/{\mathrm{cm}}^{-1}$	$\Delta E_{\mathrm{MP2}}/{\mathrm{cm}}^{-1}$
$A_{1}$	180.00	0.00	global minimum	0	0
$B_{1}$	39.74	39.74	saddle point	1274	1219
*C*	180.00	180.00	local minimum	1302	1353
*D*	0.00	0.00	saddle point	2197	2307
$E_{1}$	178.97	103.95	saddle point	2230	2284
$F_{1}$	104.69	104.69	local maximum	3197	3208
$G_{1}$	−96.05	96.05	global maximum	3566	3585

**Table 3:** Description of the low-frequency vibrational bands in the FT-far-IR spectral range. The vibrational frequencies and intensities are given in ${\mathrm{cm}}^{-1}$ and km/mol, respectively.

Mode *	Description **	Symmetry	$\nu_{\exp}$		B3LYP–D3/aug–cc–pVTZ		
			This Work	Prev. Work	$\nu_{\mathrm{harm}}$	$\nu_{\mathrm{anharm}}$	$I_{\mathrm{harm}}$
$\nu_{36}$ (17a)	O-C-C-O twisting	A”	-	202 *^b^*	190	204	1.1
$\nu_{35}$ ($\tau_{2}$)	free -OH torsion	A”	221.9	220 *^a^*	240	74	120.5
$\nu_{34}$ (17b)	O-C-C-O wagging	A”	-	295 *^b^*	296	385	3.2
$\nu_{25}$ (9a)	O-C-C-O scissoring	A’	-	307 *^a^*	309	305	4.9
$\nu_{33}$ ($\tau_{1}$)	bonded -OH torsion	A”	415.0	414 *^a^*	432	339	75.0
$\nu_{24}$ (18a)	O-C-C-O rocking	A’	-	449 *^b^*	451	442	7.1
$\nu_{32}$ (16b)	sym oop ring deformation	A”	-	456 *^b^*	463	502	0.1
$\nu_{23}$ (6b)	antisym ip ring deformation	A’	-	564 *^b^*	557	547	8.5
$\nu_{31}$ (16a)	antisym oop ring deformation	A”	-	555 *^b^*	576	550	0.2
$\nu_{22}$ (6a)	sym ip ring deformation	A’	-	581 *^b^*	591	583	2.5
$2\nu_{36}$	O-C-C-O twisting overtone	A’	-	-	382	385	-
$2\nu_{35}$	free -OH torsion overtone	A’	-	-	498	411	-
$\nu_{36,35}$	combination band	A’	-	-	441	403	-

* In parentheses, the vibrational labelling used for monosubstituted benzenes [23] and used in previous catechol studies [16,17]. ** Twisting and wagging correspond, respectively, to antisymmetric and symmetric out-of-plane (oop) bending modes; Scissoring and rocking correspond respectively to symmetric and antisymmetric in-plane (ip) bending modes. *^a^ * REMPI low-resolution gas phase vibrational measurements from [4]. *^b^ * Solid state IR and Raman measurements from [16].

**Table 4:** Summary of the fitted parameters in the far-IR analysis of the $\nu_{35}$ free -OH and $\nu_{33}$ bonded -OH torsional modes. The GS parameters were fixed to the fitted values obtained by the microwave study of Caminati et al. [11]. 1σ uncertainties are quoted in parenthesis in the units of the last digit. $N_{\mathrm{trans}}$ is the number of fitted *c*-type transitions. $N_{\mathrm{lines}}$ is the number of measured lines included in the fit. The table gives also the $J_{\max}^{\prime \prime }$ and $K_{a,\max}^{\prime \prime }$ quanta and the RMS values (in ${\mathrm{cm}}^{-1}$ and unitless).

Parameter	GS, [11]	$v_{35}$	$v_{33}$
Freq/${\mathrm{cm}}^{-1}$		221.9125852(643)	415.0091367(613)
*A*/MHz	3387.5939(1)	3385.3793(48)	3378.9268(88)
*B*/MHz	2246.1882(1)	2243.7576(32)	2246.0999(36)
*C*/MHz	1350.9738(1)	1350.96712(240)	1350.40579(106)
$\Delta_{J}$/kHz	0.091(15)	0.08983(47)	0.09726(44)
$\Delta_{K}$/kHz	0.57(1)	0.5712(33)	0.5790(91)
$\Delta_{JK}$/kHz	0.043(8)	0.04329(244)	0.0416(36)
$\delta_{J}$/kHz	0.027(1)	0.026704(270)	0.026348(218)
$\delta_{K}$/kHz	0.126(7)	0.12374(190)	0.15957(159)
$N_{\mathrm{trans}}$		6937	4131
$N_{\mathrm{lines}}$		3978	2455
$J_{\max}^{\prime \prime }$		97	199
$K_{a,\max}^{\prime \prime }$		51	41
RMS/${\mathrm{cm}}^{-1}$		0.00022	0.00023
Unitless RMS		1.10	1.17

**Table 5:** The spectroscopic constants and fit results of the mm-wave spectrum of catechol in the GS and in the lowest frequency vibrationally ES. For the rotational constants and the inertial defects $\Delta I=\frac{h}{8\pi^{2}}\left(\frac{1}{C}-\frac{1}{B}-\frac{1}{A}\right)$, the experimental fitted values are compared to the calculated values. In the lower part of the table, the number of measured lines $N_{\mathrm{lines}}$, the number of fitted transitions $N_{\mathrm{trans}}$, the maximum *J*, and $K_{a}$ values and the RMS highlighting the quality of the fit are given.

Parameter	GS	$|v_{36}=1\rangle$	$|v_{35}=1\rangle$	$|v_{34}=1\rangle$	$|v_{25}=1\rangle$
Freq/cm^−1^	-	202	221.9125852(643)	295	307
$A_{\mathrm{calc}}$/MHz **	3370.42487	3362.27296	3369.43585	3365.29996	3370.30499
$B_{\mathrm{calc}}$/MHz **	2239.43737	2238.44835	2236.82996	2240.06675	2241.95487
$C_{\mathrm{calc}}$/MHz **	1345.69441	1346.53357	1345.87423	1346.59351	1344.13595
*A*/MHz	3387.585698(280)	3379.64380(36)	3385.374399/7(130) *	3394.8332(32)	3372.6254(35)
*B*/MHz	2246.182094(175)	2245.18108(34)	2243.75593/1(124) *	2240.35362(184)	2255.98280(207)
*C*/MHz	1350.967110(157)	1351.797832(248)	1350.96338/6(91) *	1351.92941(74)	1349.34535(68)
$\Delta_{J}$/kHz	0.0796982(194)	0.080140(39)	0.079639/7(149) *	0.095688(150)	0.068570(200)
$\Delta_{K}$/kHz	0.549465(151)	0.538075(118)	0.55185/4(50) *	0.26194(196)	0.53505(157)
$\Delta_{JK}$/kHz	0.057612(78)	0.062049(116)	0.056538(48)	0.13356(65)	0.05032(86)
$\delta_{J}$/kHz	0.0280925(161)	0.0282030(141)	0.028002/3(53) *	0.036035(73)	0.023006(105)
$\delta_{K}$/kHz	0.114669(101)	0.110693(96)	0.11245(32)	0.16456(35)	0.10445(52)
$\Phi_{KJ}$/mHz	−0.0810(214)	-	-	-	-
$\phi_{J}$/μHz	2.55(130)	-	-	-	-
$\phi_{K}$/mHz	0.2445(253)	-	-	-	-
$\phi_{JK}$/mHz	0.0260(140)	-	-	-	-
$\Delta I_{\mathrm{calc}}$/${\text{amu.Å}}^{2}$ **	−0.064923	−0.76222	−0.42217	−0.4806	0.6186
*ΔI*/${\text{amu.Å}}^{2}$	−0.09344(5)	−0.77422(8)	−0.43314(29) *	−0.62657(8)	0.67186(31)
$N_{\mathrm{lines}}$	4744	2694	926 */888	395	367
$N_{\mathrm{trans}}$*a*-type	2437	1077	840 */811	384	598
$N_{\mathrm{trans}}$*b*-type	7609	4689	1687 */1622 *	670	814
$J_{\max}^{\prime \prime }$	124	129	99 */96 *	110	85
$K_{a,\max}^{\prime \prime }$	72	74	66 */66 *	53	53
RMS/MHz	0.055	0.077	0.093	0.070	0.19
Unitless RMS	1.17	1.18	0.93	1.15	1.40

* Blue numbers correspond to the transitions σ=1←σ=0 between sublevels of the splitting caused by -OH torsion tunnelling in the $v_{35}=1$ state and red numbers to the transitions σ=0←σ=1. ** Rotational constants determined by anharmonic calculations at the B3LYP/aug–cc–pVTZ level of theory.

**Table 6:** Comparison of the vibrational band centres ν and the inertia defects ΔI for the C-O oop bending and -OH torsional modes of phenol and catechol. The relative variations $\frac{\Delta \nu }{\nu }=\frac{\nu_{catechol}-\nu_{phenol}}{\nu_{phenol}}$ and $\frac{\Delta (\Delta I)}{\Delta I}=\frac{\Delta I_{catechol}-\Delta I_{phenol}}{\Delta I_{phenol}}$ from the phenol parameters issued from  [27] are given in %. $\Delta I^{*}=\Delta I_{ES}-\Delta I_{GS}$ corresponds to the inertia defect corrected by the GS value, i.e., induced by the oop vibration.

Molecule	Mode	ν/${\mathrm{cm}}^{-1}$	$\frac{\Delta \nu }{\nu }$	ΔI / ${\text{amu.Å}}^{2}$	$\frac{\Delta (\Delta I)}{\Delta I}$	$\Delta I^{*}$/${\text{amu.Å}}^{2}$	$\frac{\Delta (\Delta I^{*})}{\Delta I^{*}}$
**Phenol**	C-O oop bending	225.2		−0.57		−0.54	
**Catechol**	$\nu_{36}$ O-C-C-O twisting	202	−10%	−0.77	+35%	−0.68	+27%
**Catechol**	$\nu_{34}$ O-C-C-O wagging	295	+31%	−0.63	+11%	−0.53	−1%
**Phenol**	-OH torsion	309.1		−0.32		−0.29	
**Catechol**	$\nu_{35}$ free -OH torsion	221.9	−28%	−0.43	+34%	−0.34	+16%
**Catechol**	$\nu_{33}$ bonded -OH torsion	415.0	+34%	−0.33	+3%	−0.23	−22%

## Data Availability

The data presented in this study is available in the Supplementary Materials.

## Supplementary Materials

The following are available, a directory “*supp-mat*” of 17 files, which contains a pdf file “*note*” with a table that sums up the meaning and content of the other 16 files to be used with SPFIT/SPCAT in a single compressed directory.

## Sample Availability

Not available.

## Abbreviations

The following abbreviations are used in this manuscript:

2D scan	Two-dimensional scan
AILES	Advanced Infrared beamLine Exploited for Spectroscopy
B3LYP	Becke, 3-parameter, Lee-Yang-Parr
DFT	Density Functional Theory
ES	Excited State
FELIX	Free Electron Laser for Ir eXperiments
FT	Fourier Transform
GS	Ground State
HB	Hydrogen Bond
HF	Hartree–Fock
ip	in-plane
IR	InfraRed
MP2	Møller-Plesset perturbation theory at 2nd order
oop	out-of-plane
PES	Potential Energy Surface
REMPI	Resonance Enhanced MultiPhoton Ionisation
RMS	Root-Mean-Square
SOLEIL	Source Optimisée de Lumière d’Énergie Intermédiaire du LURE
S/N	Signal to Noise
UV	Ultra-Violet
ZBD	Zero Bias Detector
